# Developing a Machine Learning Model to Predict 180-day Readmission for Elderly Patients with Angina

**DOI:** 10.31083/j.rcm2506203

**Published:** 2024-05-31

**Authors:** Yi Luo, Xuewu Song, Rongsheng Tong

**Affiliations:** ^1^Department of Pharmacy, Sichuan Provincial People's Hospital, University of Electronic Science and Technology of China, 610072 Chengdu, Sichuan, China; ^2^Chinese Academy of Sciences Sichuan Translational Medicine Research Hospital, 610072 Chengdu, Sichuan, China

**Keywords:** readmission, machine learning, angina, elderly, predict

## Abstract

**Background:**

Readmission of elderly angina patients has become a serious problem, with a dearth of available prediction tools for readmission assessment. The objective of this study was to develop a machine learning (ML) model that can predict 180-day all-cause readmission for elderly angina patients.

**Methods:**

The clinical data for elderly angina patients was retrospectively collected. Five ML algorithms were used to develop prediction models. Area under the receiver operating characteristic curve (AUROC), area under the precision recall curve (AUPRC), and the Brier score were applied to assess predictive performance. Analysis by Shapley additive explanations (SHAP) was performed to evaluate the contribution of each variable.

**Results:**

A total of 1502 elderly angina patients (45.74% female) were enrolled in the study. The extreme gradient boosting (XGB) model showed good predictive performance for 180-day readmission (AUROC = 0.89; AUPRC = 0.91; Brier score = 0.21). SHAP analysis revealed that the number of medications, hematocrit, and chronic obstructive pulmonary disease were important variables associated with 180-day readmission.

**Conclusions:**

An ML model can accurately identify elderly angina patients with a high risk of 180-day readmission. The model used to identify individual risk factors can also serve to remind clinicians of appropriate interventions that may help to prevent the readmission of patients.

## 1. Introduction 

Angina is defined as a substernal chest pain, pressure, or discomfort [1], and is categorized as stable or unstable [2]. It is a common symptom of ischemic heart disease (IHD) and acute coronary syndrome (ACS), and a major cause of morbidity and mortality worldwide [2, 3]. The prevalence of angina increases with age in both females and males. Approximately 30,000 to 40,000 people per million are diagnosed with chronic stable angina in Western countries [2]. Approximately 4.1 million patients with coronary artery disease die each year in Europe, with 82% of these aged >65 years [4]. A study of 12,277 participants in China reported that 975 experienced angina symptoms [5].

Many angina patients are readmitted after discharge due to poor disease control. Angina after myocardial infarction has been associated with an increased risk of readmission [6]. An observational cohort study found that 11.7% of ACS patients were readmitted within 30 days [7], with most readmissions occurring primarily in elderly individuals [8]. Several studies have reported that the economic burden due to angina is increasing [9, 10, 11]. Frequent readmission may also be a difficult experience for elderly patients due to impaired mobility [12], and to the increased financial burden [13, 14]. Consequently, preventing the readmission of these patients is of major importance.

With the recent development of artificial intelligence, machine learning (ML) is increasingly being applied in the medical field [15, 16]. Indeed, several studies have reported models that predict angina-related risk [17, 18]. However, to the best of our knowledge, there is still no tool available to assess elderly angina patients for readmission. The aim of the present study was therefore to develop an ML model that predicts 180-day readmission of elderly angina patients, and to identify the important factors for readmission.

## 2. Materials and Methods

### 2.1 Study Population

This retrospective study was conducted at Sichuan Provincial People’s Hospital and included elderly patients who received inpatient treatment from July 2018 to June 2020. The inclusion criteria were: (1) age ≥60 years; (2) diagnosed with angina [19]; (3) follow-up time ≥180 days. The exclusion criteria were: (1) length of stay (LOS) <2 days or >60 days; (2) transfer to another hospital; (3) died in hospital. The age standard was determined according to the Chinese Standard for the elderly [20].

As this was a retrospective study, the requirement for informed consent was waived. The primary outcome was 180-day all-cause readmission. The personal information of patients was anonymized during the data collection process. This research was approved by the Ethics Committee of Sichuan Academy of Medical Sciences and Sichuan Provincial People’s Hospital (approval number: 2023-85).

### 2.2 Data Selection and Preprocessing

Patient information including medical records, medication information, complications, and laboratory results was collected from the hospital records system. In the case of multiple laboratory results, the most recent test results before discharge were selected. For cases with multiple readmissions, the first readmission record was used.

Data preprocessing included firstly the exclusion of variables with >90% missing data. Secondly, extreme values for laboratory results were deleted, as defined by values >3 interquartile ranges (IQR) from the end of the box. Random forest (RF) was used to replace missing values and the Z-score to standardize continuous variables. The number of medications (NOM) taken by each patient at the time of discharge was counted, and the age-adjusted Charlson comorbidity index (ACCI) was used to analyze complications.

### 2.3 Development of the Model 

Five ML algorithms were applied to develop prediction models: logistic regression (LR), k-nearest neighbor (KNN), support vector machine (SVM), gradient boosting decision tree (GBDT), and extreme gradient boosting (XGB). The data set was divided into a training set and a test set at a ratio of 7:3. The training set was applied for developing prediction models and the test set for validating model performance. Additionally, borderline synthetic minority oversampling technique (SMOTE) was used to balance readmission and non-readmission patients, and a 5-fold cross-validation method was used to evaluate the performance of the model on the training set.

### 2.4 Model Evaluation

Area under the receiver operating characteristic curve (AUROC) and area under the precision recall curve (AUPRC) were the primary indicators used to evaluate the performance of each model. The accuracy, precision, recall, and F1-value of each model were also evaluated. Furthermore, the Brier score and calibration curve were used to evaluate the calibration of models. A model was considered to have good calibration when the Brier score was ≤0.25.

### 2.5 Model Interpretation

Considering the issue of black-box in the prediction of ML algorithms, analysis by Shapley additive explanations (SHAP) is a useful tool to determine the contribution of each variable to the outputs of ML models. SHAP was therefore used in this study to quantify the contribution of each variable to the best model.

### 2.6 Statistical Analysis

All statistical analyses were performed using SPSS software version 25 (IBM SPSS Statistics, IBM Corporation, Armonk, NY, USA). The development of models was achieved with sklearn packages in Python version 3.7.0 (https://www.python.org). Counts and percentages were used for the expression of categorical variables, and these were analyzed by Chi-square test. The median (IQR) or mean ± standard deviation (SD) was used to express continuous variables. These were analyzed with the Mann-Whitney test or *t*-test. A two-sided *p* value of <0.05 was considered statistically significant.

## 3. Results

### 3.1 Study Population

The study population comprised 1502 elderly angina patients admitted to Sichuan Provincial People’s Hospital. Of these, 148 (9.85%) experienced 180-day readmission, including 93 (62.84%) males and 55 (37.16%) females. Patients were readmitted for heart failure (n = 30, 20.27%), worsened ischemic heart disease (n = 70, 47.30%), exacerbated respiratory failure (n = 22, 14.86%), or for other reasons (n = 26, 17.57%). The average age was 75.6 ± 9.3 years, and the LOS was 8 (4, 14) days. Of the 1354 patients who were not readmitted, 722 (53.32%) were males and 632 (46.68%) were females, with an average age of 72.6 ± 7.9 years, and LOS of 5 (3, 9) days. No significant differences in terms of age or LOS were observed between 180-day readmission patients and non-readmission patients. The baseline characteristics of patients are shown in Table 1.

**Table 1.** **Baseline characteristics of patients**.

Parameters		Non-Rehospitalization	Rehospitalization	*p*
		(n = 1354)	(n = 148)	
Gender				0.027
	Male	722 (53.3%)	93 (62.8%)	
	Female	632 (46.7%)	55 (37.2%)	
Age, years		72 (66, 78)	74 (68, 83)	<0.001
ACCI		4 (3, 5)	4 (3, 6)	<0.001
LOS, days		5 (3, 9)	8 (4, 14)	<0.001
NOM		8 (6, 10)	9 (8, 11)	<0.001
Coronary heart disease				<0.001
	Yes	1227 (90.6%)	146 (98.6%)	
	No	127 (9.4%)	2 (1.4)	
Myocardial infarction				0.789
	Yes	42 (3.1%)	4 (2.7%)	
	No	1312 (96.9%)	144 (97.3%)	
COPD				<0.001
	Yes	82 (6.1%)	23 (15.5%)	
	No	1272 (93.9%)	125 (84.5%)	
Hypertension				0.017
	Yes	834 (61.6%)	106 (71.6%)	
	No	520 (38.4%)	42 (28.4%)	
Diabetes				0.998
	Yes	421 (31.1%)	46 (31.1%)	
	No	933 (68.9%)	102 (68.9%)	
The number of affected vessels				0.118
	0	75 (5.54%)	2 (1.35%)	
	1	529 (39.07%)	63 (42.57%)	
	2	255 (18.83%)	24 (16.22%)	
	3	495 (36.56%)	59 (39.86%)	
Left ventricular ejection fraction		67.0 (63.0, 78.0)	66.5 (62.0, 70.0)	0.080
Laboratory results				
	Total protein, g/L	69.8 (65.2, 73.8)	67.5 (63.7, 71.2)	<0.001
	Total bile acid, µmol/L	5.4 (3.0, 9.3)	5.8 (4.2, 11.5)	0.335
	Total bilirubin, µmol/L	13.5 (10.3, 17.8)	13.3 (10.1, 16.9)	0.448
	Cholesterol, mmol/L	4.22 (3.52, 5.04)	3.77 (3.10, 4.59)	<0.001
	Lipoprotein (a), mg/L	127 (70, 279)	105 (61, 170)	0.028
	AST, U/L	26 (22, 33)	27 (22, 34)	0.487
	Osmotic pressure, mOsm/L	283 (279, 287)	283 (279, 287)	0.843
	Lactate dehydrogenase, U/L	206 (179, 249)	205 (181, 243)	0.767
	Globulin, g/L	28.9 (25.5, 32.0)	27.7 (25.1, 30.8)	0.049
	Uric acid, µmol/L	333 (274, 398)	346 (287, 432)	0.085
	Urea/Creatinine	84.2(68.7, 102.4)	75.7 (62.3, 99.2)	0.018
	Urea, mmol/L	5.92 (4.86, 7.30)	6.44 (5.01, 8.18)	0.043
	Alkaline phosphatase, U/L	79 (64, 96)	75 (62, 91)	0.165
	CKMB, U/L	12.7 (10.0, 16.2)	13.0 (9.8, 16.0)	0.829
	Creatine kinase, U/L	86 (63, 122)	78 (59, 110)	0.103
	Myoglobin, ng/mL	42.5 (31.6, 59.0)	49.0 (36.8, 77.0)	<0.001
	Creatinine, µmol/L	69.4 (58.7, 85.0)	75.2 (61.9, 99.1)	<0.001
	AST/ALT	1.20 (0.91, 1.57)	1.20 (0.93, 1.53)	0.582
	eGFR, mL/min	85.8 (71.6, 94.2)	79.8 (57.5, 91.0)	<0.001
	HS-TNTI, ng/L	4.6 (2.0, 13.0)	10.85 (3.62, 16.62)	0.002
	HDLC, mmol/L	1.22 (1.03, 1.45)	1.18 (0.97, 1.42)	0.072
	Triglyceride, mmol/L	1.50 (1.04, 2.29)	1.43 (0.93, 2.27)	0.150
	LDLC, mmol/L	2.30 (1.69, 2.92)	1.84 (1.40, 2.59)	<0.001
	Cholinesterase, KU/L	7.6 (6.5, 8.9)	7.0 (6.0, 8.3)	<0.001
	HSTNT, µg/L	0.012 (0.012, 0.012)	0.012 (0.012, 0.050)	0.443
	ALT, U/L	21 (15, 30)	22 (15, 33)	0.816
	Albumin, g/L	40.9 (37.8, 43.5)	39.4 (36.6, 41.3)	<0.001
	GGT, U/L	23 (17, 38)	24 (17, 40)	0.541
	Fibrinogen, g/L	3.08 (2.56, 3.73)	3.12 (2.58, 3.85)	0.425
	BNP, pg/mL	45.9 (20.7, 106.1)	89.7 (40.9, 207.5)	<0.001
	D-dimer, mg/L	0.38 (0.22, 0.77)	0.46 (0.27, 0.87)	0.038
	Neutrophil, ×${\mathrm{10}}^{9}$/L	4.15 (3.26, 5.24)	4.12 (3.36, 5.34)	0.978
	Blood platelet, ×${\mathrm{10}}^{9}$/L	168 (134, 206)	163 (128, 211)	0.583
	Hemoglobin, g/L	133 (122, 144)	127 (115, 139)	0.001
	HbA1c, %	6.0 (5.6, 6.8)	6.1 (5.7, 6.8)	0.314
	Eosinophil, ×${\mathrm{10}}^{9}$/L	0.10 (0.06, 0.17)	0.13 (0.07, 0.23)	0.002
	Basophil, ×${\mathrm{10}}^{9}$/L	0.028 (0.019, 0.038)	0.027 (0.018, 0.037)	0.457
	Lymphocyte, ×${\mathrm{10}}^{9}$/L	1.37 (1.06, 1.77)	1.33 (1.04, 1.61)	0.107
	Hematocrit, %	0.37 (0.04, 0.42)	0.37 (0.29, 0.41)	0.541
	Red blood cell, ×${\mathrm{10}}^{12}$/L	4.32 (3.97, 4.68)	4.23 (3.73, 4.70)	0.075
	RDW-SD, fL	44.3 (42.5, 46.6)	44.7 (41.9, 47.5)	0.305
	Monocyte, ×${\mathrm{10}}^{9}$/L	0.38 (0.29, 0.49)	0.40 (0.32, 0.48)	0.061
	hsCRP, mg/L	1.02 (0.50, 3.28)	1.14 (0.54, 5.18)	0.124
	White blood cell, ×${\mathrm{10}}^{9}$/L	6.22 (5.18, 7.56)	6.22 (5.20, 7.67)	0.803

Data presented as number (%) or range (Q1, Q3). Abbreviations: ACCI, age-adjusted Charlson comorbidity index; LOS, length of stay; NOM, number of medications; COPD, chronic obstructive pulmonary disease; AST, aspartate aminotransferase; CKMB, creatine kinase-MB; ALT, alanine aminotransferase; eGFR, estimated glomerular filtration rate; HS-TNTI, high sensitivity cardiac troponin I; HDLC, high density lipoprotein cholesterol; LDLC, low density lipoprotein cholesterol; HSTNT, high-sensitivity cardiac troponin T; GGT, γ-glutamyl transpeptidase; BNP, brain natriuretic peptide; HbA1c, glycated hemoglobin; RDW-SD, red blood cell distribution width-standard deviation; hsCRP, hypersensitive C-reactive protein.

### 3.2 Data Processing and Variable Screening

**Fig. 1.**
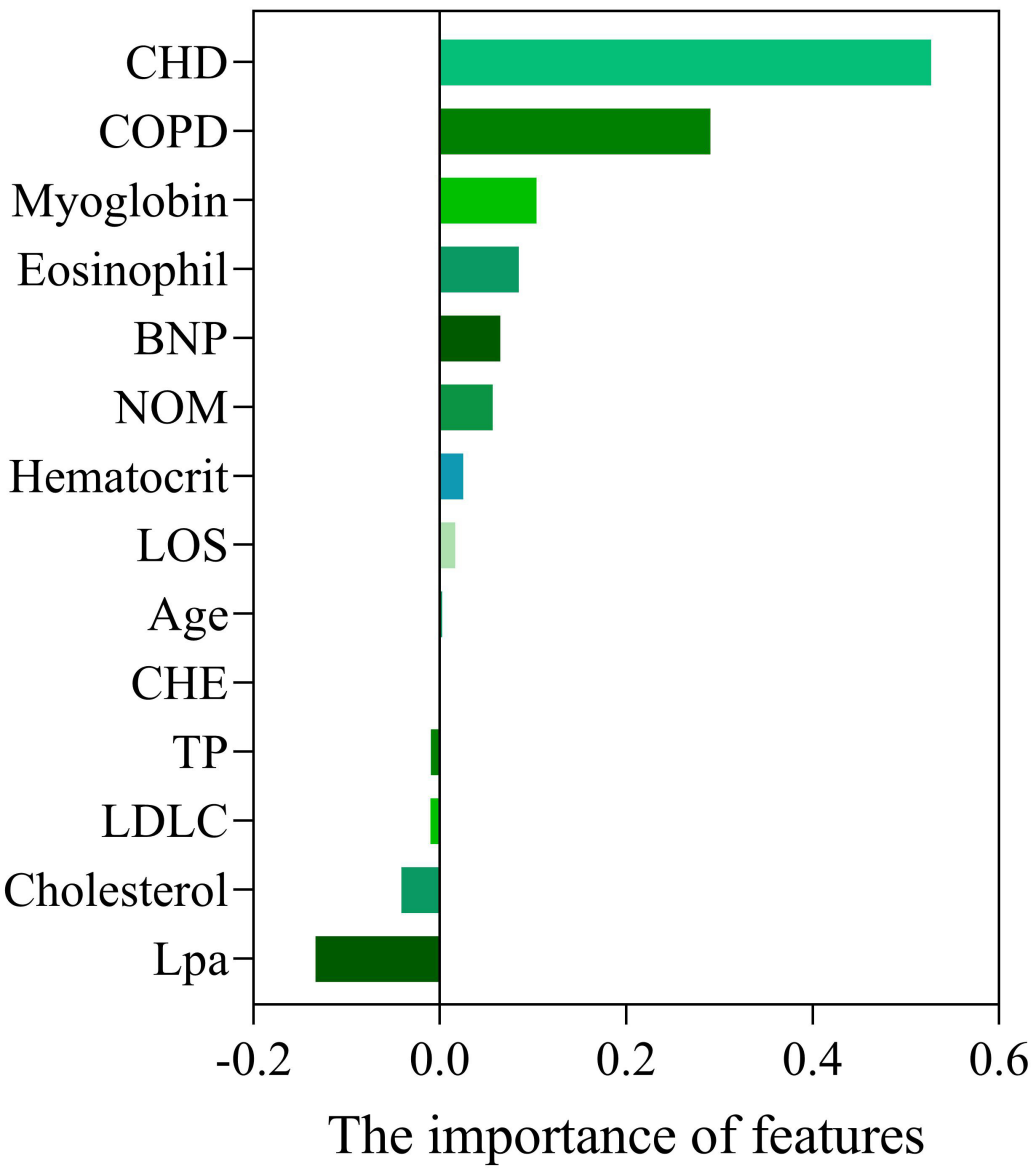
**Importance of variables selected by Lasso.** Abbreviations: CHD, coronary heart disease; COPD, chronic obstructive pulmonary disease; BNP, brain natriuretic peptide; NOM, number of medications; LOS, length of stay; CHE, cholinesterase; TP, total protein; LDLC, low density lipoprotein cholesterol; Lpa, lipoprotein (a).

Data from a total of 178 variables was collected, but 85 variables with >90% missing data were excluded. Following consultation with clinical experts, a subset of 49 variables was selected for Lasso analysis. A final set of 14 variables was chosen for the development of ML models. The importance of variables selected by Lasso is shown in Fig. 1.

### 3.3 Model Development and Evaluation

**Fig. 2.**
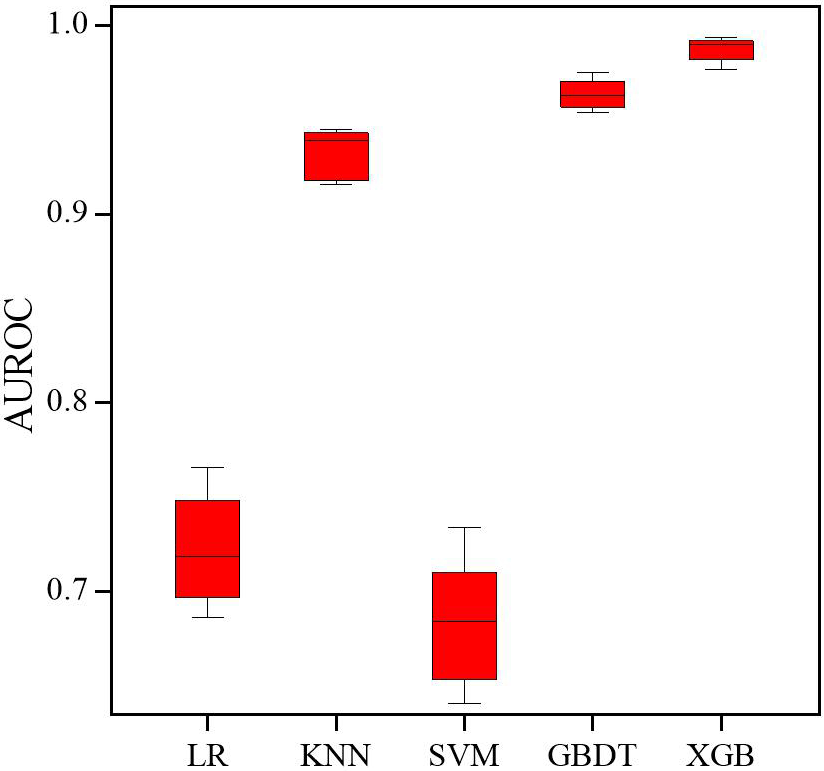
**Receiver operating characteristic (ROC) curves of the 5 machine learning models on the training set.** Abbreviations: AUROC, area under the receiver operating characteristic curve; LR, logistic regression; KNN, k-nearest neighbor; SVM, support vector machine; GBDT, gradient boosting decision tree; XGB, extreme gradient boosting.

**Fig. 3.**
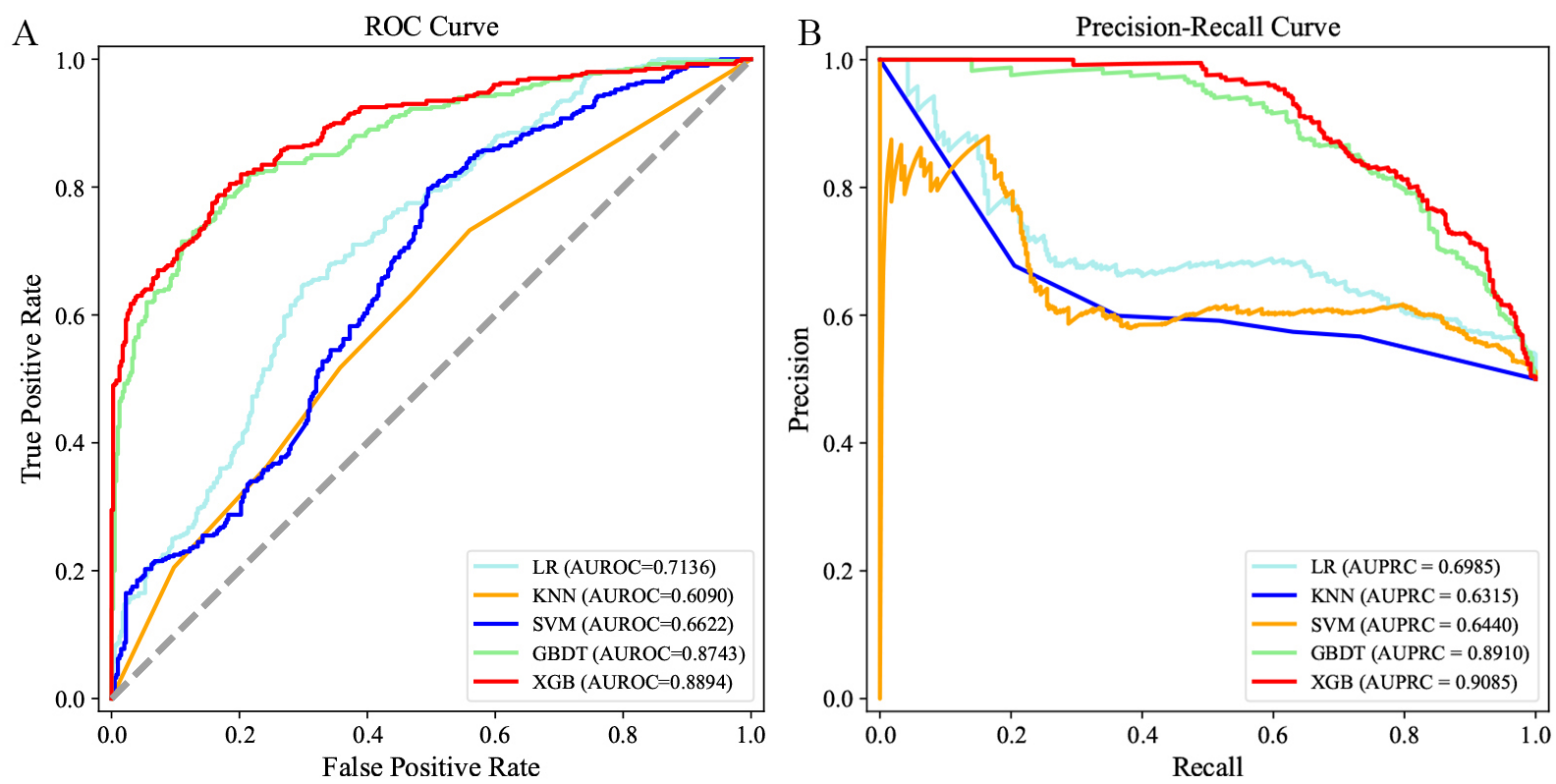
**Receiver operating characteristic (ROC) curves and precision recall curves of the 5 machine learning models on the test set.** (A) ROC curves of 180-day readmission. (B) Precision recall curves of 180-day readmission. Abbreviations: AUROC, area under the receiver operating characteristic curve; AUPRC, area under the precision recall curve; LR, logistic regression; KNN, k-nearest neighbor; SVM, support vector machine; GBDT, gradient boosting decision tree; XGB, extreme gradient boosting.

**Fig. 4.**
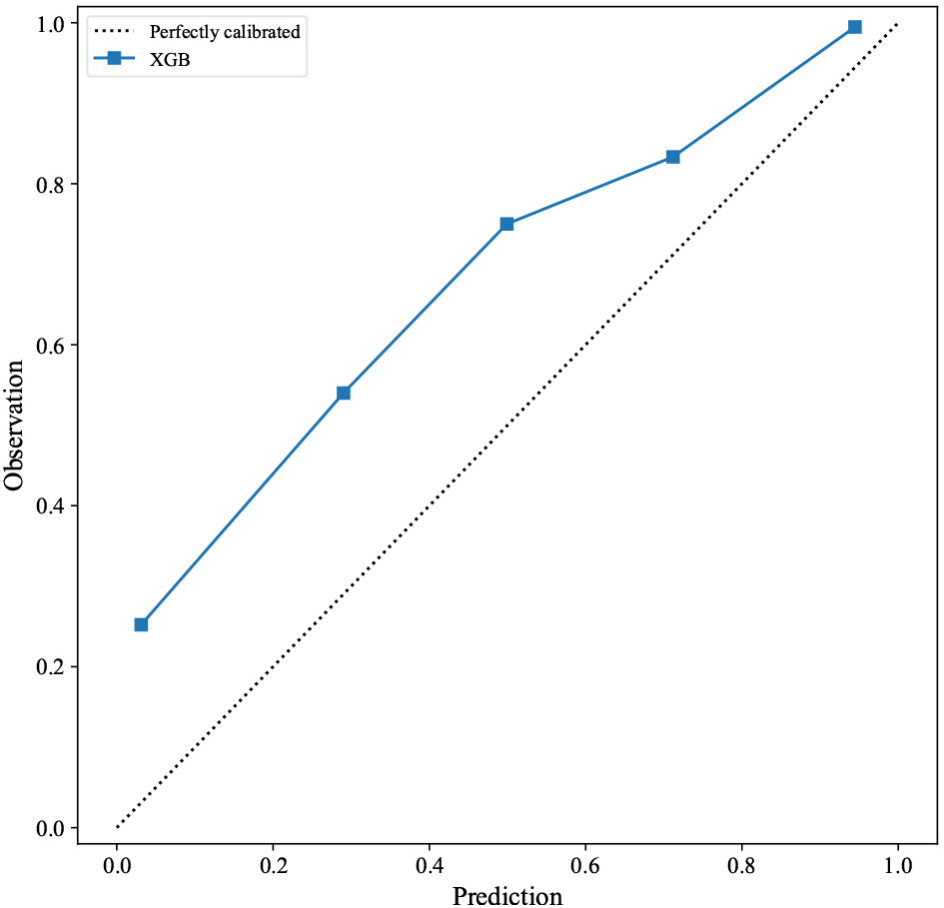
**Calibration plot of the XGB model for 180-day readmission.** Abbreviations: XGB, extreme gradient boosting.

LR, KNN, SVM, GBDT, and XGB were combined with 14 variables selected by Lasso to develop 5 models for the prediction of 180-day readmission in elderly patients with angina. The AUROC for the 5 ML models on the training set are shown in Fig. 2, with the XGB model showing the best performance. With the test set, XGB gave the best performance for AUROC (0.89), accuracy (0.79), and precision (0.96). The AUROC for the 5 ML models with the test set ranged from 0.61 (KNN) to 0.89 (XGB), while the accuracy ranged from 0.55 (SVM) to 0.79 (XGB), and the precision from 0.59 (KNN) to 0.96 (XGB). In addition, the Brier score ranged from 0.21 (XGB) to 0.45 (SVM) (Table 2). The AUROC and AUPRC for the 5 ML models used on the test set are shown in Fig. 3A,B, respectively. We also evaluated the calibration ability of the XGB model, since this model had the best predictive performance in the test set. The XGB model was found to have good calibration ability (Fig. 4).

**Table 2.** **The predictive performance of models with the test set**.

Model	Accuracy	Precision	Recall	F1-value	Brier score
LR	0.66	0.67	0.69	0.66	0.34
KNN	0.58	0.59	0.52	0.55	0.42
SVM	0.55	0.60	0.27	0.38	0.45
GBDT	0.78	0.88	0.64	0.74	0.22
XGB	0.79	0.96	0.60	0.74	0.21

Abbreviations: LR, logistic regression; KNN, k-nearest neighbor; SVM, support vector machine; GBDT, gradient boosting decision tree; XGB, extreme gradient boosting.

### 3.4 Model Interpretation

**Fig. 5.**
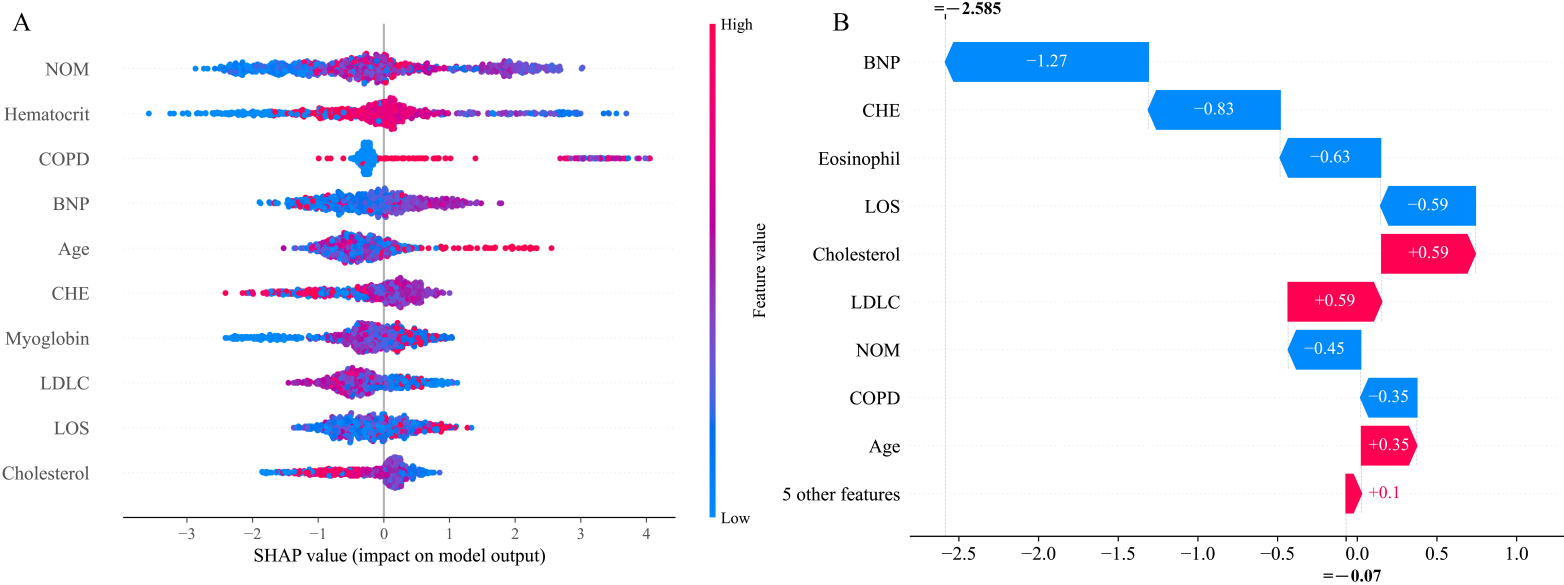
**Contributions of input variables to readmission predictions.** (A) The SHAP summary plot of the 10 most important variables of the XGB model for 180-day readmission. (B) Contribution of every variable to the predicted outcome of one sample. Red represents positive contribution, and blue represents negative contribution. Abbreviations: NOM, number of medications; COPD, chronic obstructive pulmonary disease; BNP, brain natriuretic peptide; CHE, cholinesterase; LDLC, low density lipoprotein cholesterol; LOS, length of stay; SHAP, Shapley additive explanations.

SHAP values were calculated to determine the contribution of each variable to the prediction results. As shown in Fig. 5A, the 6 most important contributors to the predictions were NOM, hematocrit, chronic obstructive pulmonary disease (COPD), brain natriuretic peptide (BNP), age, and cholinesterase (CHE).

The contribution of each variable to the predicted outcome of individual patients was also determined using illustrative examples (Fig. 5B). In the case shown, a positive effect on the prediction result was provided by cholesterol, low density lipoprotein cholesterol (LDLC), and age, whereas a negative effect was provided by BNP, CHE, eosinophil, LOS, NOM, and COPD.

## 4. Discussion

Five ML algorithms combined with 14 variables were used in this study to develop prediction models for the risk of 180-day readmission in elderly angina patients. The XGB model was found to have the best predictive performance, as shown by the highest AUROC and AUPRC values. Furthermore, the XGB model exhibited good calibration performance, as demonstrated by the Brier score and calibration curve.

Although several models for predicting angina-related risk have been reported [21, 22, 23], there are no readily available tools for predicting all-cause readmission of individual, elderly angina patients. Several studies have evaluated readmission following cardiovascular disease (CVD) [24, 25]. Okere *et al*. [24] developed a decision tree model to predict 30-day hospital readmission of IHD patients, with their model showing good predictive performance. Another study used 5 ML algorithms to predict 30-day all-cause readmission in a cohort of 1962 patients with CVD. This decision tree model showed a high F1-value (64%), precision (57%), and recall (71%) [25].

Compared to the aforementioned models, the ML model developed in the present study was more accurate and convenient. Moreover, the two previous models lacked external validation and calibration evaluation, thus making them unsuitable for assessment of readmission in elderly angina patients. Although the AUROC in our model was slightly lower than that of Okere *et al*. [24] (0.89 vs 0.95), it was developed specifically for elderly angina patients, and is therefore more appropriate for readmission assessment in this population. Additionally, the Brier score combines model discrimination and calibration, and represents the mean square error between the predicted and observed results. When 2 models are compared, a smaller Brier score indicates better model performance. In the current study, the Brier score for the XGB model was lower than that of the other ML models. Hence, the XGB model exhibited good calibration ability for predicting 180-day all-cause readmission in elderly angina patients.

Many clinical variables have been associated with readmission in the existing literature [7, 26, 27, 28, 29, 30, 31, 32, 33]. To increase the accuracy of prediction models, it is important to identify the significant influencing factors from amongst the many complex factors in elderly angina patients. After considering the potential influence of common clinical variables on the prediction results, 14 variables were selected from 178 clinical features in order to develop a practical model. Elderly patients often experience polypharmacy due to multiple comorbidities, which may increase the risk of adverse drug events [34]. The present study confirmed CHD and NOM as predictors in the models. Consistent with our findings, previous studies have reported that age, BNP, and LOS affect hospital readmission for cardiovascular patients [29, 30, 33]. In our study, age, BNP, and LOS were associated with an increased risk of 180-day readmission. Furthermore, we also identified other important factors for the readmission of elderly angina patients, including hematocrit, LDLC, and cholesterol. Interestingly, the relationship between readmission and some of the variables revealed by SHAP analysis in our study is not immediately consistent with clinical intuition. However, these variables may still reflect the extent of patient illness, thus helping to predict outcome. Another advantage of this study is that SHAP was used to determine individual risk, which may help in providing optimal patient care and appropriate intervention.

Nevertheless, there are several limitations to this study. Firstly, the single-center study design means that analysis of data from other medical institutions is needed to test the predictive performance of our model. Secondly, this study collected only common clinical data. Further research is therefore required to determine the influence of other factors that could positively impact the readmission of patients with cardiac diseases, such as continuity of care, self-care, and perceived control [35, 36]. Thirdly, although complications in elderly patients were incorporated into this research, relevant information such as disease severity and the duration of complications were not included. Finally, a prospective study is required to verify the clinical utility of our prediction model.

## 5. Conclusions

This study developed models to predict 180-day all-cause readmission in elderly angina patients by combining 5 ML algorithms with common clinical variables. The XGB model demonstrated superior predictive performance compared to the other models. This ML-based tool could have future clinical applications for the identification of 180-day readmissions in elderly angina patients, as well as for improving their quality of care.

## Data Availability

The datasets used and/or analyzed during the current study are available from the corresponding author on reasonable request.
